# Spontaneous intraperitoneal rupture of pyonephrosis in a patient with unknown kidney carcinosarcoma: a case report

**DOI:** 10.1186/1477-7819-9-39

**Published:** 2011-04-12

**Authors:** Silvia Quaresima, Antonio Manzelli, Edoardo Ricciardi, Athanasios Petrou, Nicholas Brennan, Alessandro Mauriello, Piero Rossi

**Affiliations:** 1 Cattedra di Chirurgia Generale, Università degli Studi di Roma Tor Vergata

## Abstract

Seventeen cases of peritonitis due to rupture of a pyonephrosis have been reported. The majority of these cases occur secondary to renal stones. Only two cases of ruptured pyonephrosis with concurrent kidney neoplasm have been described and only one of these presented as an acute peritonitis. In this presentation we discuss an unusual case of a 68 year old man with a chronic history of bilateral nephrolithiasis and recent pyonephrosis. He presented acutely with peritonitis and was later found to have a carcinosarcoma of the kidney. The case highlights the importance of recognizing the possibility of underling renal carcinoma in patients presenting with a ruptured pyonephrosis and discuss steps to avoid this serious complication.

## Background

Peritoneal fistulization of a pyonephrosis is an extremely rare event which invariably leads to generalized peritonitis [1]. Rupture of a pyonephrotic kidney is usually associated with a previous kidney abnormality with hydropyonephrosis or pyonephrosis a common precipitator. Renal stones and, much less commonly, neoplasms may also cause rupture [2]. The renal origin of peritonitis is more often revealed intraoperatively as the clinical condition of the patient does not allow full urological investigation before laparotomy [3].

The aim of this paper is to present an unusual case of a 68 years old man, with a previous history of gallstone pyonephrosis, presenting with an acute abdomen and having a final diagnosis of renal carcinosarcoma.

## Case presentation

A 68 years old man with a chronic history of bilateral nephrolithiasis was admitted to our department with high grade fever, rigors, lower back and diffuse abdominal pain. His past medical history included insertion of a left urethral stent six months earlier for pyonephrosis. The double J stent had not resolved the hydronephrosis and was due to be changed in the coming weeks. There was no history of diabetes mellitus. On examination, temperature was 39.5°C, heart rate 103 beats per minute (b.p.m), respiratory rate 28 breaths per minute, blood pressure 178/88 mmHg and there was generalized abdominal guarding and rigidity. White Blood Cells (WBC) were 24.600/cu mm, C-reactive protein (CRP) >160 mg/L, Haemoglobin (Hb) 9.1 gr/dl and Lactate Dehydrogenase (LDH) 220.000 Ul/l. No pre-operative urine or blood cultures were performed.

**Figure 1:**
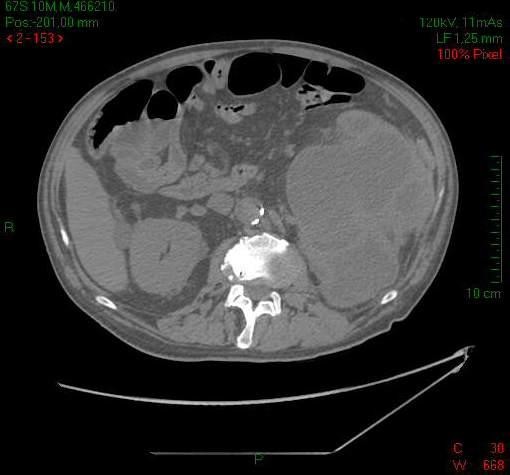
**CT image depicting a grossly enlarged left kidney contained within the Gerota capsule**.

**Figure 2:**
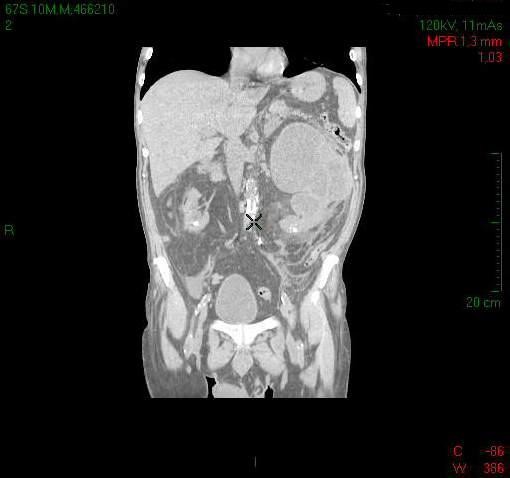
**CT image depicting a grossly enlarged left kidney contained within the Gerota capsule**.

An abdominal computed tomography (CT) scan was performed which revealed massive distension of the left kidney contained within the Gerota capsule. There was severe distension of the ascending/transverse colon and the left sided intestinal loops with a minor fluid collection in the pelvis but no free air (Figure 1 &2). The patient proceeded to an explorative laparotomy which indeed revealed a purulent peritonitis. The left kidney had the appearance of a large sac, containing an abundance of pus which leaked through a small fistula in the overlying adherent peritoneum into the peritoneal cavity. This fluid was sent for culture. As a result of these findings a left nephrectomy was performed.

**Figure 3:**
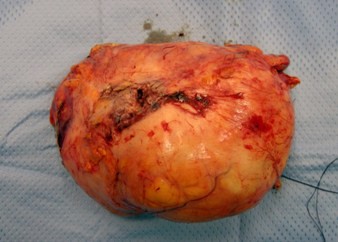
**Surgical specimen of the resected left kidney with surrounding oedema and suffusion of perinephric fat tissue**.

**Figure 4:**
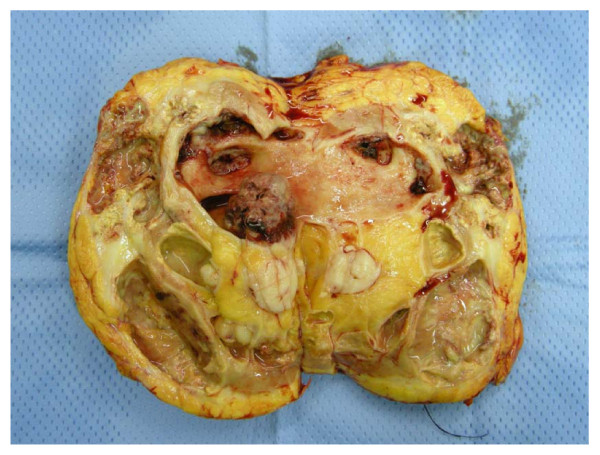
**Surgical specimen of the resected left kidney with surrounding oedema and suffusion of perinephric fat tissue**.

**Figure 5:**
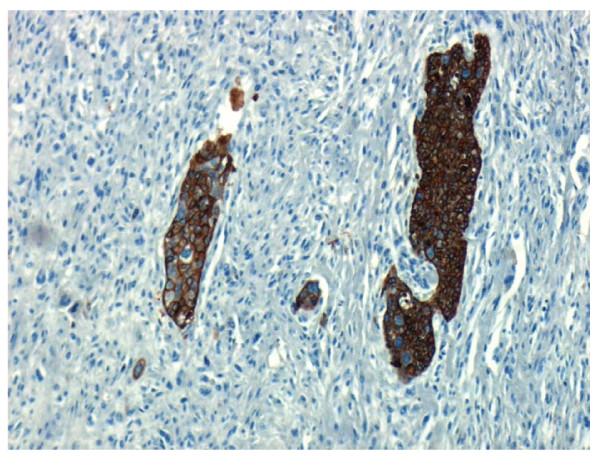
**Histology speciment with cytokeratin stain**.

**Figure 6:**
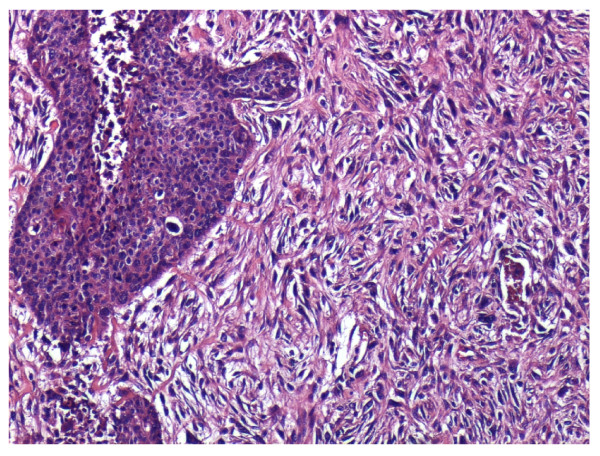
**Histology speciment with Ematossilin-Eosin preparation**.

**Figure 7:**
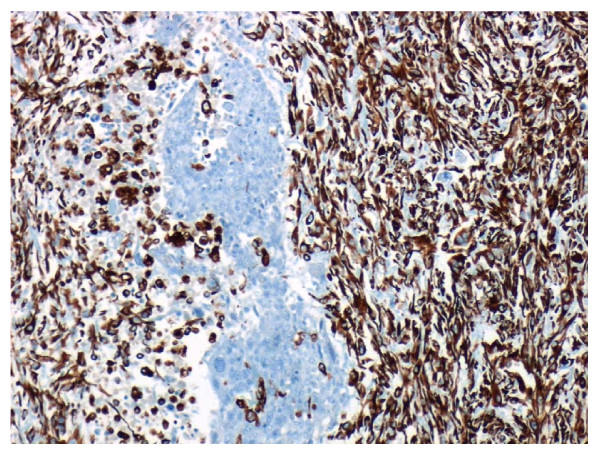
**Histology speciment with Vimblastine preparation**.

Analysis of the kidney specimen revealed a 20 cm × 11 cm mass with a gelatinous centre and pus filled cysts in the renal pelvis (Figure 3 &4). The histopathology report documented a pelvis carcinosarcoma of the left kidney with a staging pT4, Nx, Mx. Figures 5, 6, and 7 demonstrate the histology specimens with cytokeratin stain, Ematossilin-Eosin and Vimblastine preparations respectively. It is important to mention perirenal tissue infiltration was from the retroperitoneal side rather than posterior muscular abdominal wall. Postoperatively, the patient had an uneventful recovery and was referred to the oncology team. Based on the clinical signs of sepsis and peritonitis pre-operatively, and the microbiological profile of the cultured intraabdominal pus, the patient received a course of post-operative antibiotics. The oncological team performed the indicated postoperative restaging, including a new MDCT scan, and MRI scan, which failed to demonstrate the existence of distant metastatic disease. The patient underwent a multi-agent chemotherapy, and, radiation therapy. On the oncological follow-up, 11 months postoperatively, the patient remains alive and in reasonably good clinical condition with his most recent imaging negative for disease recurrence.

## Discussion

Rupture of the kidneys more commonly occurs at the site of the renal parenchyma over the renal pelvis. In these instances, hemorrhage is at the forefront. This may be limited to the subcapsular space, confined in the renal fossa by the circumrenal fascia, or so massive to involve one side of the abdomen, engulfing the kidney in a shell of hemorrhagic tissue. In contrast urinary extravasation without hemorrhage is characteristic of a ruptured pelvis, although secondary hemorrhage can often occur. If rupture was preceded by a pyonephrosis the extravasated material may be infected. Dispersion may be limited by Gerota's capsule or involve the retroperitoneum and this may lead to fistulization into the abdominal cavity [4]. Abeshouse *et al *remarked that spontaneous pelvic rupture practically always occurs in a kidney which is seat of chronic pylonephritis or where there is dilatation secondary to obstruction [5]. Mathe *et al *included the additional conditions; stone formation, chronic nephritis, tuberculosis, abscess formation, infarct, aneurysm and tumor [6]. Miller and Kaufmann reviewed the cases of spontaneous kidney rupture with associated neoplasms and noted less than 50 patients [7]. These included: hypernephroid carcinoma, angiomyolipoma, transitional cell renal carcinoma, Wilm's tumor, angiosarcoma, liposarcoma, fibrosarcoma and papillary carcinoma of the renal pelvis. Over 70% of these tumours contained sarcomatoid elements [7].

Sarcomatoid renal cell carcinoma (SRCC), first described by Farrow et al. in 1968, is defined pathologically by highly pleomorphic spindle cells and/or giant cells resembling sarcoma, with varying degrees of clear or granular epithelial cells that characterize SRCC [8]. A sarcomatoid component is indicative of an aggressive tumour [9-11]. These tumors are usually symptomatic at the time of diagnosis and often cause haematuria, abdominal pain and a mass in the flank [12-14]. Radiologically there are few specific signs which differentiate these tumours from other renal carcinomas [15]. The majority of cases present in an advanced stage with renal capsule invasion or distant metastases, most commonly to lung and bone [13-17]. Treatment involves nephrectomy with the addition of adjuvant therapies such as radiotherapy, chemotherapy and immunotherapy.

In the literature, only two cases of kidney neoplasm with a background of recurrent pyonephrosis have been reported and only one of these presented as an acute peritonitis. There have been seventeen cases of peritonitis due to rupture of a pyonephrosis: seven derived from spontaneous rupture of pyonephrosis in patients with urolithiasis or hydronephrosis and one in a patient with renal tuberculosis [18-23]. Two uncommon cases of peritonitis secondary to rupture of a retroperitoneal abscess and another from rupture of an infected urachal cyst have also been described [24,25]. Only one other paper reports the presentation of peritonitis due to a kidney neoplasm: Bittard *et al *discuss the case of a 44 year old man presenting with an acute abdomen. Laparotomy revealed a stercoraceous peritonitis from diastatic colic rupture with a T4 kidney tumor infiltrating the right colon [26].

Intraperitoneal rupture of a pyonephrosys is a rare event which needs immediate intervention. The clinical presentation is of an acute abdomen with increased inflammatory markers and occasionally an associated pleural effusion. The predominant abdominal symptoms mask the underlying renal cause, and the peritonitis is attributed to intestinal perforation or appendicitis. X-rays fail to demonstrate free air in the diaphragm, although in rare cases they can highlight radiopaque stones. CT scanning is certainly the most sensitive at demonstrating the presence of underlying renal disease and subsequent laparotomy is inevitable. Establishing the fistulous site may not be possible due to inflammation with adhesions between parietal peritoneum and omentum. A careful exploration of the peritoneal cavity and all intestinal tracts is therefore necessary.

In conclusion, this report highlights the importance of recognizing the possibility of underlying renal carcinoma in patients presenting with peritonitis and a history of pyonephrosis and stresses the significance in early and full urological investigation to avoid severe complication.

## Consent

Written informed consent was obtained from the patient for publication of this case report and any accompanying images. A copy of the written consent is available for review by the Editor-in-Chief of this journal.

## Competing interests

The authors declare that they have no competing interests.

## Authors' contributions

SQ, AM, PR, ER and GP made up the surgical and pathological team invovled in the case. AP and NB wrote and edited the manuscript. All authors read and approved the final manuscript.
